# Role of *FOXC2* and *PITX2* rare variants associated with mild functional alterations as modifier factors in congenital glaucoma

**DOI:** 10.1371/journal.pone.0211029

**Published:** 2019-01-18

**Authors:** Cristina Medina-Trillo, José-Daniel Aroca-Aguilar, Jesús-José Ferre-Fernández, Susana Alexandre-Moreno, Laura Morales, Carmen-Dora Méndez-Hernández, Julián García-Feijoo, Julio Escribano

**Affiliations:** 1 Área de Genética, Facultad de Medicina, Universidad de Castilla-La Mancha, Albacete, SPAIN; 2 Instituto de Investigación en Discapacidades Neurológicas (IDINE), Universidad de Castilla-La Mancha, Albacete, SPAIN; 3 Cooperative Research Network on Prevention, Early Detection and Treatment of Prevalent Degenerative and Chronic Ocular Pathology (OftaRed), Instituto de Salud Carlos III, Madrid, SPAIN; 4 Servicio de Oftalmología, Hospital San Carlos, Madrid, SPAIN; 5 Instituto de Investigación Sanitaria del Hospital Clínico San Carlos, Madrid, SPAIN; University of Iowa, UNITED STATES

## Abstract

Congenital glaucoma (CG) is a severe and inherited childhood optical neuropathy that leads to irreversible visual loss and blindness in children. CG pathogenesis remains largely unexplained in most patients. Herein we have extended our previous studies to evaluate the role of *FOXC2* and *PITX2* variants in CG. Variants of the proximal promoter and transcribed sequence of these two genes were analyzed by Sanger sequencing in a cohort of 133 CG families. To investigate possible oligogenic inheritance involving *FOXC2* or *PITX2* and *CYP1B1*, we also analyzed *FOXC2* and *PITX2* variants in a group of 25 CG cases who were known to carry *CYP1B1* glaucoma-associated genotypes. The functional effect of three identified variants was assessed by transactivation luciferase reporter assays, protein stability and subcellular localization analyses. We found eight probands (6.0%) who carried four rare *FOXC2* variants in the heterozygous state. In addition, we found an elevated frequency (8%) of heterozygous and rare *PITX2* variants in the group of CG cases who were known to carry *CYP1B1* glaucoma-associated genotypes, and one of these *PITX2* variants arose *de novo*. To the best of our knowledge, two of the identified variants (*FOXC2*: c.1183C>A, p.(H395N); and *PITX2*: c.535C>A, p.(P179T)) have not been previously identified. Examination of the genotype-phenotype correlation in this group suggests that the presence of the infrequent *PITX2* variants increase the severity of the phenotype. Transactivation reporter analyses showed partial functional alteration of three identified amino acid substitutions (*FOXC2*: p.(C498R) and p.(H395N); *PITX2*: p.(P179T)). In summary, the increased frequency in PCG patients of rare *FOXC2* and *PITX2* variants with mild functional alterations, suggests they play a role as putative modifier factors in this disease further supporting that CG is not a simple monogenic disease and provides novel insights into the complex pathological mechanisms that underlie CG.

## Introduction

Primary congenital glaucoma (PCG; MIM# 231300) is a severe and irreversible neonatal or infantile optic neuropathy of uncertain pathogenesis. The immature iridocorneal angle appearance observed clinically indicates that it results from arrested maturation of tissues derived from cranial neural crest cells. This alteration leads to increased aqueous humor (AH) outflow resistance, elevated intraocular pressure (IOP) and optic nerve degeneration. The diagnosis is based in finding isolated trabeculodysgenesis with no other ocular anomalies [1]. PCG is the most common non-syndromic glaucoma in infancy [2], occurring in one of 10,000–20,000 live births in Western countries [3], and it presents increased incidence in consanguineous populations [4, 5]. PCG leads to significant visual loss and blindness in children. Autosomal-recessive inheritance is well documented in this disease, mainly due to *CYTOCHROME P450*, *SUBFAMILY I*, *POLYPEPTIDE 1* (*CYP1B1*, MIM# 601771) mutations [6]. Absence of a family history and variable penetrance (ranging from 40% to 100% [6]) is reported in most cases, although a family history is more common in consanguineous families. Classical linkage analysis has identified loss-of-function variants in *CYP1B1* [7] and *LATENT TRANSFORMING GROWTH FACTOR-β-BINDING PROTEIN 2* (*LTBP2*, MIM# 602091) [8] as the cause of the disease in some patients. Recently, whole-exome sequencing revealed that mutations in the angiopoietin receptor *TEK* (*TEK*, MIM# 600221) underlie PCG with variable expressivity [9] and that rare and hypermorphic *G-PATCH DOMAIN-CONTAINING PROTEIN 3* (*GPATCH3*, MIM# 617486) variants are present in a some congenital glaucoma (CG) cases [10]. Null *CYP1B1* mutations are the predominant known genetic cause of this type of glaucoma in different worldwide populations [7, 11–13], and *LTBP2* gene alterations have been identified only in a few families [8, 14, 15]. *MYOCILIN* (*MYOC*, MIM# 601652) [16, 17] and *FORKHEAD BOX C1* (*FOXC1*, MIM# 601090) alterations [18, 19] have also been found in a small number of CG cases. The genetic heterogeneity present in PCG, along with the frequent incomplete penetrance and variable expressivity, even in patients with null *CYP1B1* genotypes [13], strongly indicates the participation of modifier genetic and/or environmental factors in the pathogenicity of this type of glaucoma. Previous studies from our laboratory have provided evidence for the role of rare *FOXC1* variants with moderate functional dysregulation as either causative of modifier factors in CG [18–20].

FORKHEAD BOX C2 (*FOXC2*, MIM# 602402) and *PAIRED-LIKE HOMEODOMAIN TRANSCRIPTION FACTOR 2* (*PITX2*, MIM# 601542), are genes encoding transcription factors that control the expression of other genes and form part of a complex regulatory network involved in ocular development and disease [21]. *FOXC2* is structurally and functionally closely related to *FOXC1*. Their chromosomal localization and genomic organization suggest a common origin from an ancestral gene through chromosomal duplications [22, 23]. The proteins encoded by these two genes share 98% sequence identity at their forkhead domain with only two amino acid differences [24], and present overlapping expression patterns during embryonic development, including similar expression in the periocular mesenchyme and in periocular mesenchyme-derived tissues involved in formation of the AH drainage pathway [25–27]. Either a complete loss or a significant gain of *FOXC2* function cause lymphedema-distichiasis syndrome, an autosomal dominant disease that affects the formation of the lymphatic vasculature system and is also characterized by a double row of eyelashes (distichiasis) [28].

The *PAIRED-LIKE HOMEODOMAIN TRANSCRIPTION FACTOR 2* (*PITX2*, MIM# 601542) gene encodes a homeodomain-containing transcription factor. It presents a pattern of expression similar to that of *FOXC1* and *FOXC2*, and it is also present in the periocular mesenchyme. Disruption of *PITX2* is one of the major causes of Axenfeld-Rieger syndrome (ARS) [29]. ARS is a clinically and genetically heterogeneous group of developmental dominant disorders, affecting primarily the anterior segment of the eye, frequently associated with secondary glaucoma [30]. Systemic alterations, such as dental defects, mild craniofacial dysmorphism and umbilical anomalies may also be present in ARS patients. ARS is a fully penetrant disease with variable expressivity. *PITX2*, like *FOXC1*, is dose sensitive and mutations that alter the level of functional PITX2 protein (either increased or decreased) by different mechanisms are pathogenic [30]. The *PITX2* gene is composed of six exons, two alternative promoters located upstream of exons 1 and 4, and two alternative transcription start sites located in exons 2 and 4 [31]. The immature mRNA is alternatively spliced to produce four isoforms (PITX2A-D). The first three isoforms share an identical homeodomain and C-terminal region, and they differ in their N-terminal sequences [31].

In this study we show an increased frequency of rare and functionally altered *FOXC2* and *PITX2* variants in CG patients, providing further evidence for the role of rare variants of these two genes involved in ocular anterior segment development as putative modifier factors.

## Materials and methods

### Subjects

One hundred and thirty-three unrelated families affected by PCG participated in this study. The study and informed written consent procedures were approved by the Ethics Committee for Human Research of the Hospital Clínico San Carlos, Madrid (Spain), approval number 13/388-E) and followed the tenets of the Declaration of Helsinki. The clinical examination and diagnosis of patients were performed as previously described [13, 19]. Glaucoma was ruled out in 100 control individuals, who were recruited among patients diagnosed with cataracts, floaters, refractive errors or itchy eyes.

### Variant screening

The genomic DNA was extracted from peripheral blood, using the *QIAamp DNA Blood Mini Kit* (Qiagen, Valencia, CA, USA). The DNA sequence variation analyses were carried out by automatic Sanger sequencing. The promoter region (nucleotides -1 to -1500), the coding region and both the 5'- and 3’-untranslated regions (UTRs) of *FOXC2* were amplified by PCR, using the primers and conditions described in S1 Table. The coding region, 5'- and 3'-UTRs of *PITX2* were amplified as previously described [19]. The variants identified in each subject were confirmed by sequencing a new amplification product. The patients with rare *FOXC2* variants were screened for *LTBP2* mutations using a customized gene panel [32]. Library capture of all exons and 20 bp of intronic boundaries was performed using SureSelect QXT technologies (Agilent Technologies, Santa Clara, CA, United States). Massive sequencing was carried out using Illumina MiSeq or NextSeq 500 platforms running on paired-end mode at a minimum of 450X. Bioinformatic analysis was performed using standard procedures and custom in-house pipelines for mapping, variant calling and annotation.

### Cloning and site directed mutagenesis of *FOXC2* and *PITX2* variants

The *FOXC2* variants p.(C498R) (rs61753346) and p.(H395N) were directly amplified and cloned from the genomic DNA of carriers using the following primers, which incorporated the *Eco*RI or *Bam*HI restriction sites at the 5’ end (indicated in bold): FOXC2-Up-*Eco*RI, 5'-GG**GAATTC**CGCGCTCTCTCGCTCTCAGG-3' and FOXC2-Dw-*Bam*HI, 5'-GG**GGATCC**CCGTATTTCGTGCAGTCGTAGG-3'. The PCR products were cloned into a modified version of the *Eco*RI/*Bam*HI restriction sites of the pcDNA3.1(-) vector (C-terminal myc-tagged) [33]. The same approach was used to clone the wild-type *FOXC2* coding sequence from DNA of a control subject and the cloned DNA was completely sequenced to rule out the presence of rare FOXC2 variants. The *PITX2* coding variants p.(P179T) was obtained by site-directed mutagenesis using the QuickChange Site-Directed Mutagenesis Kit (Agilent, Santa Clara, CA, USA), with the following primers (mutant nucleotides are indicated in bold): F, 5’-CCTACGACGACATGTAC**A**CAGGCTATTCCTACAAC-3’ and R, 5’-GTTGTAGGAATAGCCTG**T**GTACATGTCGTCGTAGG-3’. A cDNA encoding the wild-type PITX2C (NP_000316) isoform, cloned into the pcMV6 vector (Origene, NM_000325), was used as a template for the site-directed mutagenesis reaction. The presence of the mutation in the site-directed mutagenesis product was confirmed by Sanger sequencing. The mutagenized cDNA was subcloned into the *Sgf*I and *Mlu*I restriction sites of pcMV6 to avoid undesirable mutations in the vector.

### Transactivation luciferase reporter assays

Transactivation assays were performed using the Luciferase Assay System (Promega) following the instructions of the manufacturer. HEK-293T cells were transfected with the different recombinant versions of FOXC2 and PITX2C, cloned in the expression vector pcMV6 (500 ng each), along with the recombinant pGL3-basic-*CXCR4* luciferase reporter vector (50 ng), and the pMirTarget vector (50 ng), which expresses the red fluorescent protein (RFP) as a transfection control. The cells were cultured in Dulbecco’s modified Eagle’s medium (DMEM) supplemented with 10% fetal bovine serum (FBS) and antibiotics (Normocin, Invivogen), at 37°C in a fully humidified 5% CO_2_ atmosphere. Forty-eight hours after transfection the cells were harvested and assayed for firefly luciferase activity using the Luciferase Assay System (Promega) according to the manufacturer’s instructions. FOXC2 and PITX2 proteins were detected by Western blot as expression control and transactivation activity was normalized for these proteins. RFP and endogenous lactate dehydrogenase (LDH) were also assessed by Western blot as transfection and loading controls, respectively.

### Protein stability and half-life calculation

The protein stability was studied via western blot of transfected cells treated with cycloheximide (300 μg/ml) at different times as previously reported [34]. The FOXC2 and PITX2 protein levels were determined by densitometry and the relative amounts at different time points after cycloheximide treatment were expressed as a percentage of the levels at time 0 h. At least three independent assays for each variant were performed. The transfection efficiency was assessed by co-transfecting the recombinant cDNA constructs encoding the different mutants (500 ng) with the pMirTarget vector (50 ng), which encodes the red fluorescent protein (RFP). The RFP was detected via Western blotting. The loaded samples were normalized for total protein content using the Bradford reagent (Pierce, Rockford, IL, USA). FOXC2 and PITX2 decay followed a first order kinetics. The slope of the decay line was calculated using standard linear regression, and the protein half-life was determined as previously described [35].

### Western blotting and antibodies

For Western blot analyses the cell lysates were prepared and fractionated using sodium dodecyl sulfate-polyacrylamide gel electrophoresis (SDS-PAGE), using the Mini-PROTEAN III Gel Electrophoresis System (BioRad, Hercules, CA, USA). A commercial mouse monoclonal anti-myc (sc-40, Santa Cruz Biotechnology, Santa Cruz, CA, USA) was used as the primary antibody, diluted at 1:1000. Horseradish peroxidase-conjugated antibodies against mouse IgG (#32439, Invitrogen) were diluted to 1:1000–1:4000. Chemiluminiscence detection and the densitometry for protein band quantification was performed as previously described [36]. As an additional sample loading control, the endogenous lactate dehydrogenase (LDH) protein was detected in cell extracts using a goat anti-LDH antibody diluted to 1:5000 (AB1222, Chemicon, Temecula, CA, USA) and an anti-goat IgG horse-radish peroxidase-conjugated antibody (sc-2033, Santa Cruz Biotechnology, diluted to 1:2000). RFP (transfection control) was detected using a rabbit anti-RFP antibody (#AB233, Evrogen, Moscow, Russia), diluted to 1:5000, and an anti-rabbit IgG horse-radish peroxidase-conjugated antibody (#1858415, Pierce), diluted to 1:1000.

### Bioinformatic analyses

The deleterious effect of mutations was predicted online with the Sorting Intolerant From Tolerant (SIFT) (Kumar et al., 2009) and PolyPhen-2 (http://genetics.bwh.harvard.edu/pph2/) programs. The *MicroSNiPer* software was used to identify putative miRNA target sequences (http://cbdb.nimh.nih.gov/microsniper) (Barenboim et al., 2010). Both nucleotide and amino acid sequence alignments were carried out with Clustalw (Thompson et al., 1994). The novel FOXC2 and *PITX2* variants were named using directions from Mutalyzer (https://humgenprojects.lumc.nl/trac/mutalyzer), according to RefSeq NM_005251.2 and NM_000325.5, respectively. The novel variants have been submitted to dbSNP (http://www.ncbi.nlm.nih.gov/SNP/) and to ClinVar (https://www.ncbi.nlm.nih.gov/clinvar/). Structure prediction of mutant and wild-type proteins were evaluated by QUARK (https://zhanglab.ccmb.med.umich.edu/QUARK2/) [37] and further visualized using Pymol Molecular Graphics System (Pymol).

### Statistical analysis

The statistical comparisons between groups were performed using either the *t*-test or the one-way analysis of variance (ANOVA). A Bonferroni correction was applied to adjust tests for multiple comparisons. The data were statistically processed by the SigmaStat 2.0 software (SPSS Science Inc., Inc., Chicago, IL, USA).

## Results

### *FOXC2* and *PITX2* variants

**Fig 1:**
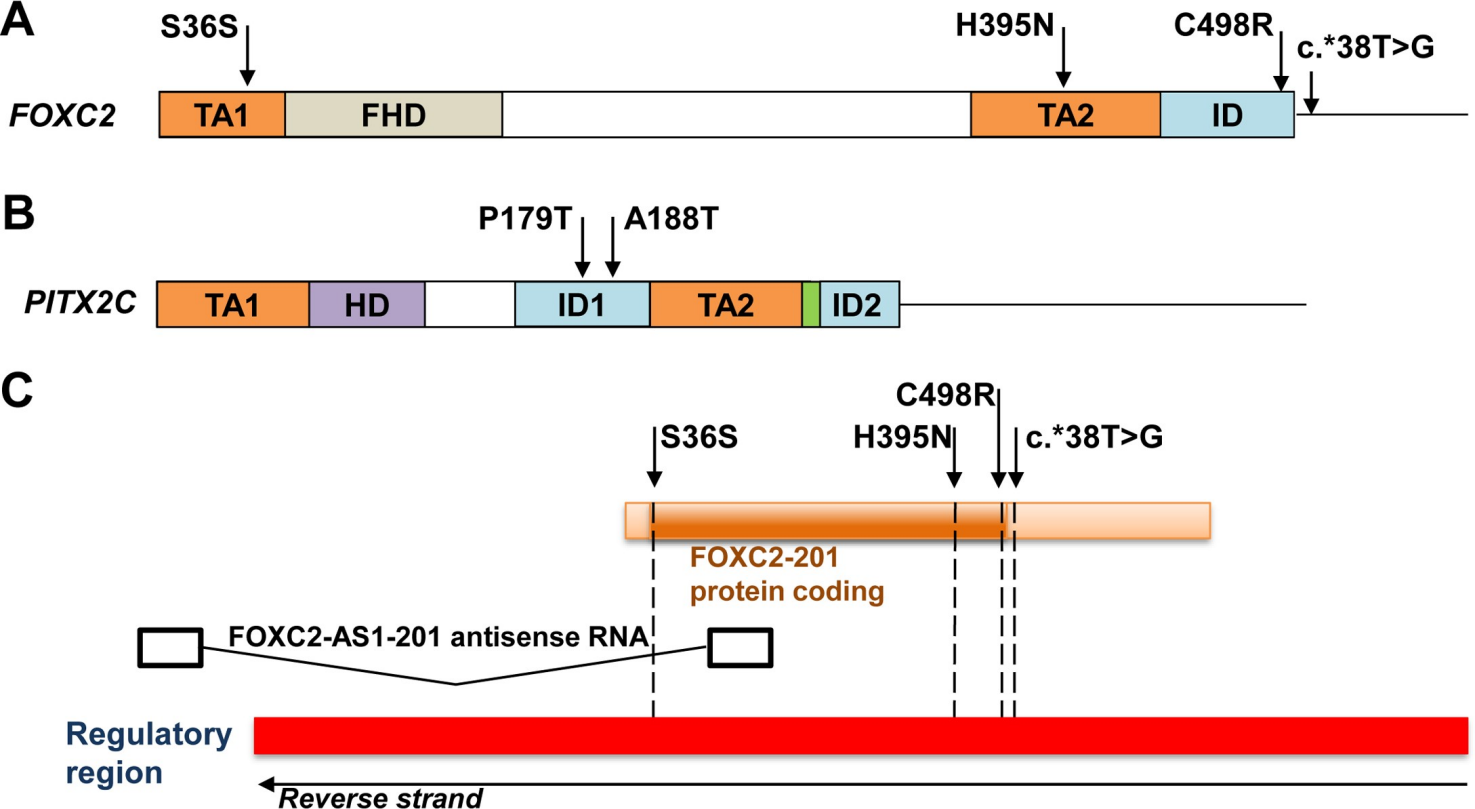
Location of *FOXC2* and *PITX2* variants identified in congenital glaucoma patients in this study. (A, B) Cartoons correspond to *FOXC2* and *PITX2C* mRNAs indicating the localization of different encoded protein domains. Rectangles the solid lines represent coding and 3’UTR sequences, respectively. FHD, forkhead domain; HD: homeodomain; ID, inhibitory domain; TA, transactivation domain. The green box in *PITX2C* mRNA indicates the 14-aminoacid OAR (Otp and ARistaless) domain [39]. (C) *FOXC2* and *FOXC2-AS1* gene structure as a notated in Ensembl. Note the presence of a promoter (regulatory region, red rectangle) overlapping the two genes.

**Fig 2:**
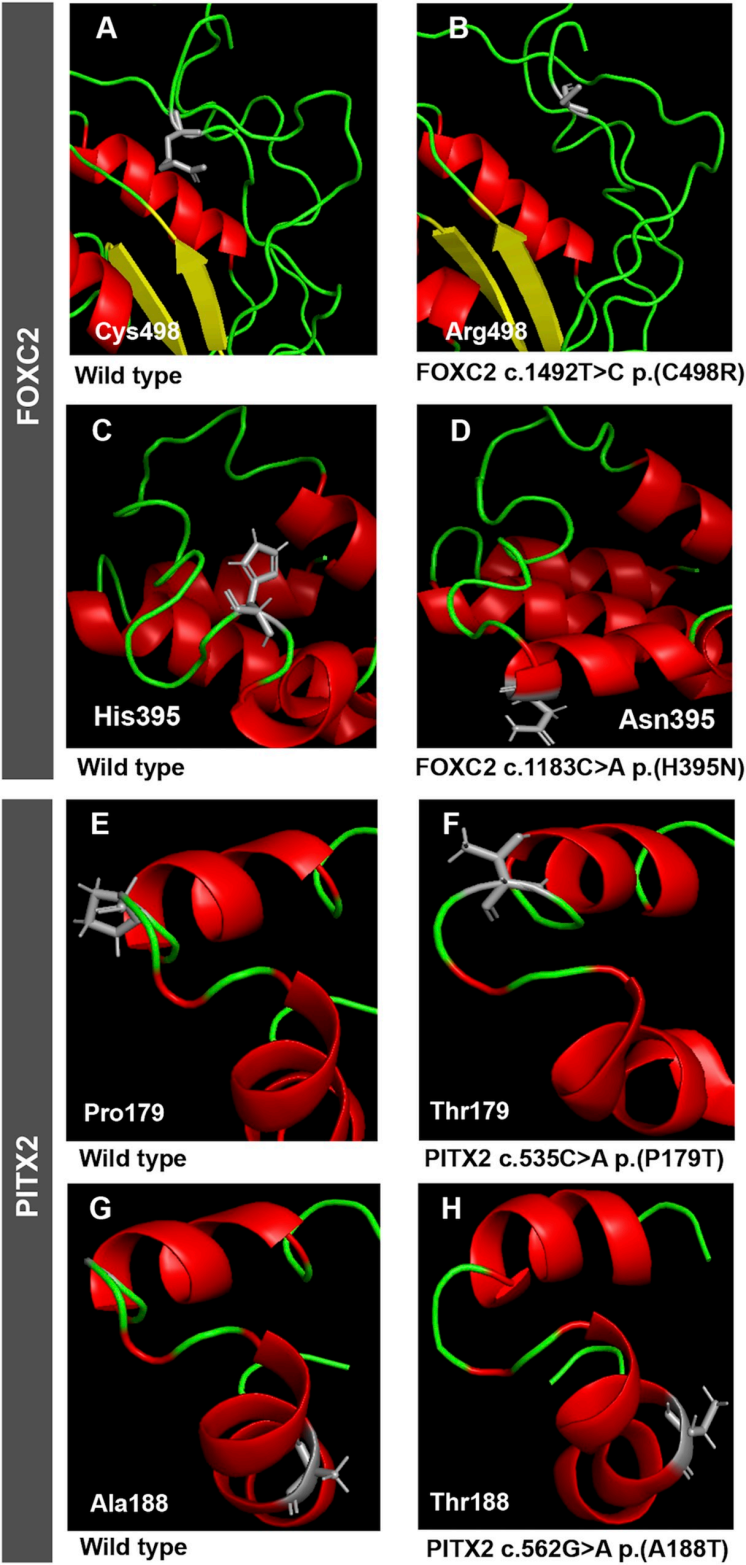
Prediction of conformational changes induced by the identified coding *FOXC2* and *PITX2* variants. Predicted model structures of wild type (left) (A, C, E and G) and mutant (right) proteins (B, D, F and H) were obtained with QUARK as described in Materials and Methods.

To further investigate the role in CG of variants of genes involved in ocular development we have extended our previous genetic studies on a large cohort of 133 apparently unrelated PCG families, in which we reported an increased frequency of rare and functionally altered *FOXC1* variants [18, 20]. In the present work we evaluated the role of *FOXC2* and *PITX2* variants in CG using the same group of patients. The main clinical and demographic features of these patients have previously been reported [19]. These patients had no known disease-causing variants in the coding regions of *MYOC* and *CYP1B1*. Eight of the patients (6.0%) carried rare *FOXC2* variants in the heterozygous state (Table 1 and S1 Fig) and in contrast, none of them presented rare *PITX2* variants. Rare *FOXC1* and *FOXC2* variants were not coinherited in any of these patients. In addition, the presence of pathogenic *LTBP2* variants was ruled out in these subjects by Next Generation Sequencing. All the *FOXC2* carriers identified in this study had bilateral PCG and most of them were diagnosed before the fourth month of life and required several surgical interventions for adequate control of IOP (Table 2). Segregation analyses of the identified variants did not show a monogenic pattern of co-inheritance with glaucoma, and these variants were not detected in 100 Spanish control individuals (200 chromosomes). The p.(C498R) (rs61753346) substitution was found in two probands (PCG-190 and PCG-143) with different age at diagnosis and evolution of glaucoma (Table 2). Patient PCG-190 was diagnosed with PCG at birth, his left eye was eviscerated and required two surgical operations for adequate control of IOP. However, patient PCG-143 was diagnosed at the age of five years and his IOP was well controlled with medication. This variant was located in the inhibitory domain of the protein (Fig 1A) and its predicted effect evaluated with the Ensembl Variant Effect Predictor [38] ranged from moderate to deleterious, and its reported frequency in 1000 Genomes and ExAC databases was lower than 0.1% (Table 1). Moreover, structural prediction using QUARK showed that cysteine 498 is located in a disordered region of the protein and although its substitution by arginine is inferred to cause weak conformational disruption (Fig 2A and Fig 2B), the presence of a positive charge in this position of the mutant polypeptide chain may affect chemical interactions and protein function. Interestingly, this variant and the rest of *FOXC2* nucleotide changes identified in this study, overlapped a regulatory feature (ENSR00000089180), which is annotated in Ensemble and GeneCards databases as a promoter covering the entire *FOXC2* sequence and most of the antisense lncRNA *FOXC2-AS1* gene (Fig 1C). The synonymous change p.(S36S) (rs138318843) was carried by four patients (3%), showing a 4- to 10-fold increased frequency compared with that reported in 1000 Genomes and ExAC databases (0.3% and 0.7%, respectively, Table 1). The variant was associated with variable age at diagnosis and IOPs, ranging from two and a half months to three years and from 22 to 45 mmHg, respectively (Table 2). p.(S36S) carriers required different surgical procedures for correct IOP control (Table 2). This nucleotide substitution also mapped at *FOXC2-AS1* intron 1 (n.145+174G>A) and the regulatory feature (promoter) (Fig 1C), and it was inferred to produce a low functional effect on *FOXC2* and a modifier outcome on both *FOXC2-AS1* and the overlapping promoter. The nucleotide change c.*38T>G was located in the 3’UTR of the gene (Fig 1A) and was identified in one patient diagnosed at the age of two and a half months, who required several trabeculectomies to correct IOP (Table 2). This variant had a frequency of 0.1–0.2% in the two genome databases used in the study and was bioinformatically classified as a modifier nucleotide substitution (Table 1) in the two overlapping genomic elements (Fig 1C), andaccording with microSNIPER it does not affect microRNA targets. The last identified *FOXC2* nucleotide change was predicted to cause a novel amino acid change (p.(H395N)) located in the second transactivation domain of the protein (Fig 1A) with inferred moderate functional effect (Table 1). The structural prediction showed that the substitution of histidine 395 with asparagine in the polypeptide chain increases the length of an alpha helix (Fig 2B and Fig 2C). In addition, the bioinformatic analysis indicated that it was a modifier variant for the overlapping *FOXC2-AS1* gene and the promoter (Table 1 and Fig 1C). The patient who carried this variant was diagnosed at the age of three months, showed severe optic nerve excavation and was subjected to one goniotomy in the right eye and two trabeculectomies in the left eye (Table 2).

**Table 1:** *FOXC2*, *PITX2* and *CYP1B1* variants identified in congenital glaucoma patients.

Family	Variant^a^	%, 1000 Genomes/ExAC (number of homozygotes)	Functional assay	EVEP/Polyphen^b^/SIFT^c^
PCG-143 PCG-190	FOXC2^d^: c.1492T>C, p.(C498R) (rs61753346)	0.03/0.094 (1)	hypomorphic	FOXC2: Moderate/Probably damaging (0.987)/deleterious (0.01) Promoter^e^: modifier/-/-
PCG-209	FOXC2: c.*38T>G (rs199552394)	0.2/0.1 (1)		FOXC2: modifier/-/- Promoter: modifier/-/-
PCG-8 PCG-36 PCG-163 PCG-171	FOXC2: c.108C>T, p.(S36S) (rs138318843) FOXC2-AS1^f^: n.145+174G>A	0.3/0.7 (5)		FOXC2: low/-/- FOXC2-AS1: modifier/-/- Promoter: modifier/-/-
PCG-150	FOXC2: c.1183C>A, p.(H395N)	N.I.	hypomorphic	FOXC2: moderate/benign (0.19)/tolerated (0.06) Promoter: modifier/-/-
PCG-133	PITX2^g^: c.535C>A, p.(P179T) (de novo). CYP1B1: c.535delG/c.1159G>A, A179fs*18/E387K	N.I.	hypermorphic	NA/benign/not tolerated (0.01)
PCG-139	PITX2: c.562G>A, p.(A188T) (rs77144743) CYP1B1: c.1159G>A/c.517G>T, E387K/E173*	0.1/0.36 (2)		Missense/beningn (0.02)/ tolerated (0.61)

^a^All variants were present in the heterozygous state.

^b^Polyphen scores: 0.0–0.15, benign; 0.15–1.0, possibly damaging; 0.85–1.0, damaging.

^c^Threshold for SIFT intolerance is 0.05.

^d^*FOXC2* RefSeq: NM_005251.2.

^e^Ensembl transcript ID: ENSR00000089180 (regulatory feature).

^f^Ensembl *FOXC2-AS1* transcript ID: ENST00000563280.1.

^g^PITX2 RefSeq: NM_000325.5; PITX2C amino acid sequence was used as a reference to number coding variants (NP_000316).

N.I.: Not Identified; EVEP: Ensembl Variant Effect Predictor. NA: not available. -: not apply.

**Table 2:** Rare *FOXC2* and *PITX2* gene variations identified in PCG and associated clinical features.

Proband (family member)	Nucleotide change	Amino acid change	Laterality/Sex	Age at diagnosis	IOP (mmHg) at diagnosis (OD/OI)	C/D (OD/OS)	Number and type of surgery (OD/OS)
PCG-143	FOXC2^a^: c.1492T>C (rs61753346)	p.(C498R)	B/F	5y/F	21/21	0.5/0.1	No surgery
PCG-190	FOXC2: c.1492T>C (rs61753346)	p.(C498R)	B/M	0m/M	NA/EE	0.5/EE	No surgery/EE
PCG-209	FOXC2: c.*38T>G (rs199552394)	-	B/M	2.5m	26.5/22	NA (leukoma)	3T/2T
PCG-8	FOXC2: c.108C>T (rs138318843) FOXC2-AS1^b^: n.145+174G>A	FOXC2: p.(S36S)	B/M	2.5m	22/22	NA	CTT/CTT
PCG-36	FOXC2: c.108C>T (rs138318843) FOXC2-AS1: n.145+174G>A	FOXC2: p.(S36S)	B/M	4m	NA	NA	G/G
PCG-163	FOXC2: c.108C>T (rs138318843) FOXC2-AS1: n.145+174G>A	FOXC2: p.(S36S)	B/F	3m	45/40	0.8/0.7	G+T/3G
PCG-171	FOXC2: c.108C>T (rs138318843) FOXC2-AS1: n.145+174G>A	FOXC2: p.(S36S)	B/M	3y	NA	0.9/1.0	T/T
PCG-150	FOXC2: c.1183C>A	p.(H395N)	B/M	3m	NA	0.8/0.5	G+T/T
PCG-133 (II:1)	PITX2^c^: c.535C>A (de novo). CYP1B1: c.535delG/c.1159G>A	PITX2: p.(P179T) CYP1B1: A179fs*18/E387K	B/F	3m	24/22	0.8/0.8	G+2T+/2G+3T
PCG-133 (II:2)	CYP1B1: c.535delG/c.1159G>A	A179fs*18/E387K	B/M	10y	NA	NA	NA
PCG-139	PITX2: c.562G>A (rs77144743). CYP1B1: c.1159G>A/c.517G>T	PITX2: p.(A188T) CYP1B1: E387K/E173*	B/M	0m	NA	0.4/0.5	2G+T/EE

B/U: bilateral/unilateral; CTT/G/T: combined trabeculotomy-trabeculectomy/goniotomy/trabeculectomy; F/M: female/male; m/y: months/years; NA: not available; OD/OS: right eye/left eye; EE: eviscerated eye

^a^*FOXC2* RefSeq: NM_005251.2.

^b^Ensembl *FOXC2-AS1* transcript ID: ENST00000563280.1.

^c^*PITX2* RefSeq: NM_000325.5; PITX2C amino acid sequence was used as a reference to number coding variants (NP_000316).

### Coinheritance of *PITX2* and *CYP1B1* rare variants

**Fig 3:**
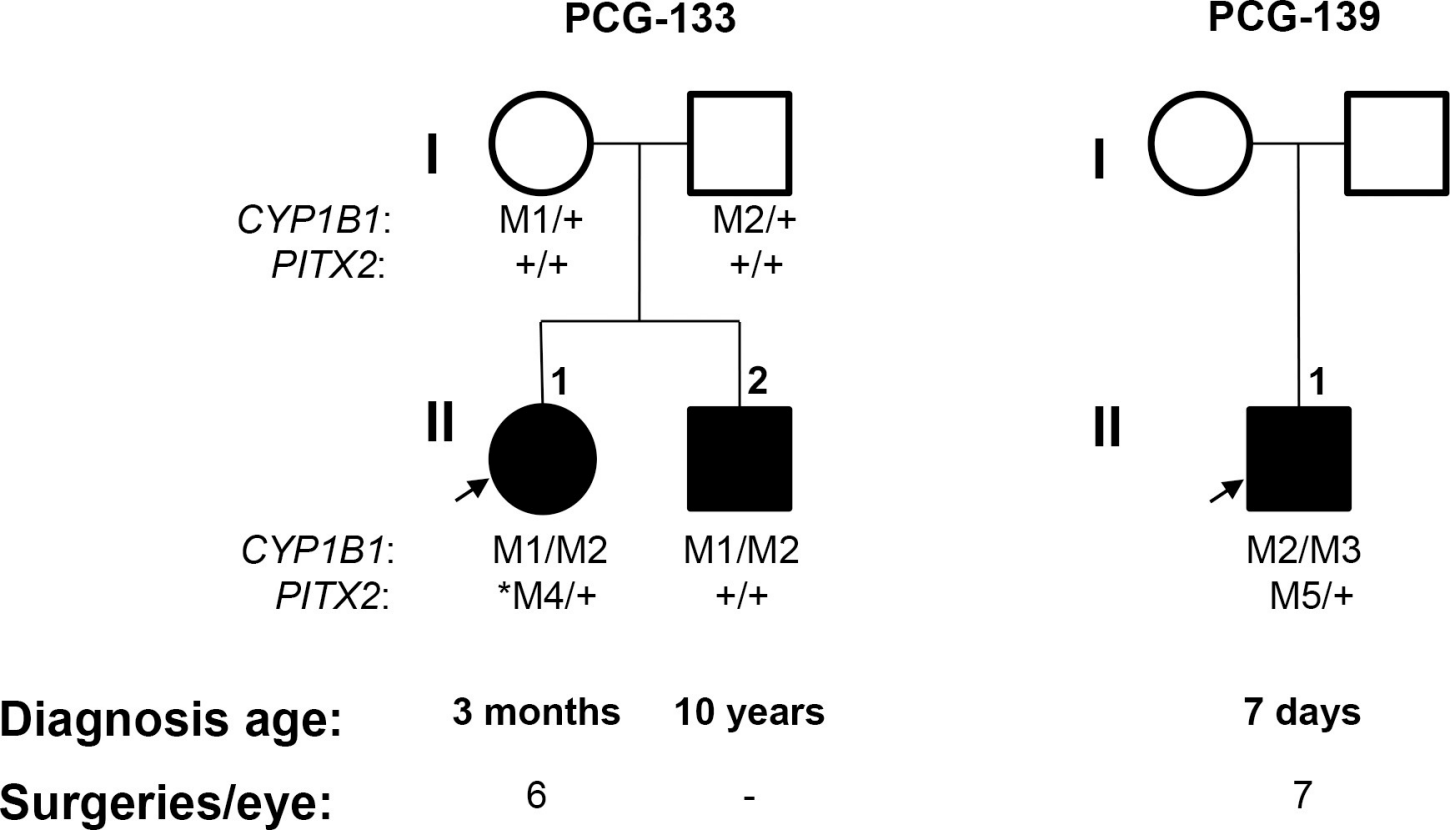
Evidence for modifier effect of rare *PITX2* variants on age at onset and severity in *CYP1B1* congenital glaucoma patients. All mutations were detected in the heterozygous state. Note that subject II:1 in family PCG-133 was diagnosed at the age of 3 months and carried the *de novo* p.(P179T) *PITX2* variant, whereas his brother, who did not carry this variant, was diagnosed at the age of 10 years. The proband in family PCG-139 also carried a rare *PITX2* variant (p.(A188T)) and presented glaucoma diagnosed at the age of seven days. Both probands required more surgical operations to control IOP than the rest of patients. Below symbols are indicated genotypes for *CYP1B1* and *PITX2*, age at diagnosis and number or surgical operations per eye, respectively. M1, *CYP1B1*: p.(A179fs*18). M2, *CYP1B1*: p.(E387K). M3, *CYP1B1*: p.(E173*). M4, *PITX2*: p.(P179T). M5, *PITX2*: p.(A188T). Arrows show the index cases. +: wild-type allele. The asterisk indicates a *de novo PITX2* variant.

We also evaluated the possible modifier role of *FOXC2* and *PITX2* defects on patients with *CYP1B1* glaucoma-associated genetic backgrounds. To that end, a group of 25 CG cases who were known to carry *CYP1B1* pathogenic genotypes, were selected based on DNA availability from a previously reported cohort of patients [13]. Two heterozygous *PITX2* variants were identified in two (8%) of these patients (PCG-133 and PCG-139). As far as we know, one of these variants (c.535C>A, p.(P179T), S1 Fig) was novel, was located in a transcriptional inhibitory domain (Fig 1B) and segregation analysis revealed that although the progenitors were heterozygous carriers for the *CYP1B1* variants none of them presented this *PITX2* nucleotide change (Fig 3), indicating that it was acquired *de novo*. The patient who carried this variant (PCG-133) was diagnosed with bilateral PCG at the age of three months and presented advanced optic nerve excavations and was subjected to six surgical operations per eye to control IOP (Table 2). Interestingly, her brother, who inherited the same *CYP1B1* genotype and did not present the *PITX2* variant, was diagnosed with bilateral infantile glaucoma at the age of ten years. The predicted amino acid substitution was classified as not tolerated by SIFT, although it was considered benign by Polyphen (Table 1). The structural prediction showed no significant conformational changes (Fig 2E and Fig 2F), although the different chemical properties of the mutant amino acid might affect PITX2 molecular interactions. Altogether, the data indicate that this *de novo PITX2* variant is a possible genetic modifier of glaucoma onset. The second *PITX2* variant identified among *CYP1B1* deficient patients was a coding nucleotide substitution (c.562G>A), that resulted in an inferred missense mutation (p.(A188T)), (S1 Fig) also located in the first transcriptional inhibitory domain (Fig 1B). This nucleotide change had a frequency of 0.1–0.36% in the two databases used in this study (Table 1). The variant was classified as benign or tolerated by the bioinformatic analyses and structural prediction indicated that the conformation of the protein is not significantly disrupted by this amino acid change (Fig 2G and Fig 2H). The carrier of this combined genotype (subject PCG-139) was diagnosed with severe bilateral PCG at birth, which required from seven to eight surgical operations per eye to achieve an adequate IOP control. The number of glaucoma surgical operations of the two *PITX2* was higher than the average for other patients. These data suggest a modifier effect of this rare *PITX2* variant on age at onset and severity in *CYP1B1* congenital glaucoma patients.

### Evolutionary conservation of *FOXC2* and *PITX2* variants

**Fig 4:**
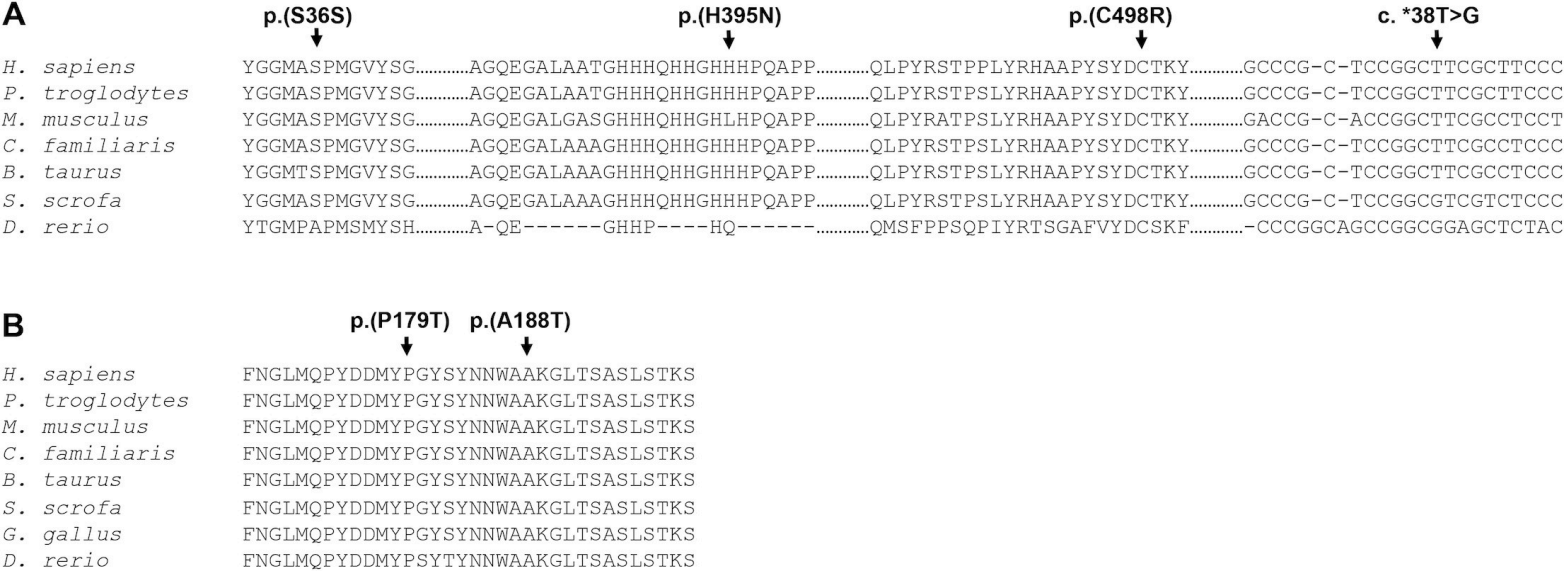
Multiple amino acid and nucleotide sequence alignment of FOXC2 and PITX2 from different vertebrate species. Sequence alignment was generated by ClustalW (http://www.ebi.ac.uk/clustalw/). Residues affected by mutations are indicated by arrows.

Evolutionary amino acid or nucleotide sequence conservation analysis were assessed using a multiple sequence alignment of representative orthologous proteins or genes of seven different species, from fish to human. This analysis revealed that most *FOXC2* and *PITX2* wild-type amino acids or nucleotides were highly conserved (Fig 4). These data indicate that the identified variants affect functionally important positions, and therefore that they may affect the function of these genes and the corresponding proteins.

### Transactivation activity, protein stability and subcellular localization

**Fig 5:**
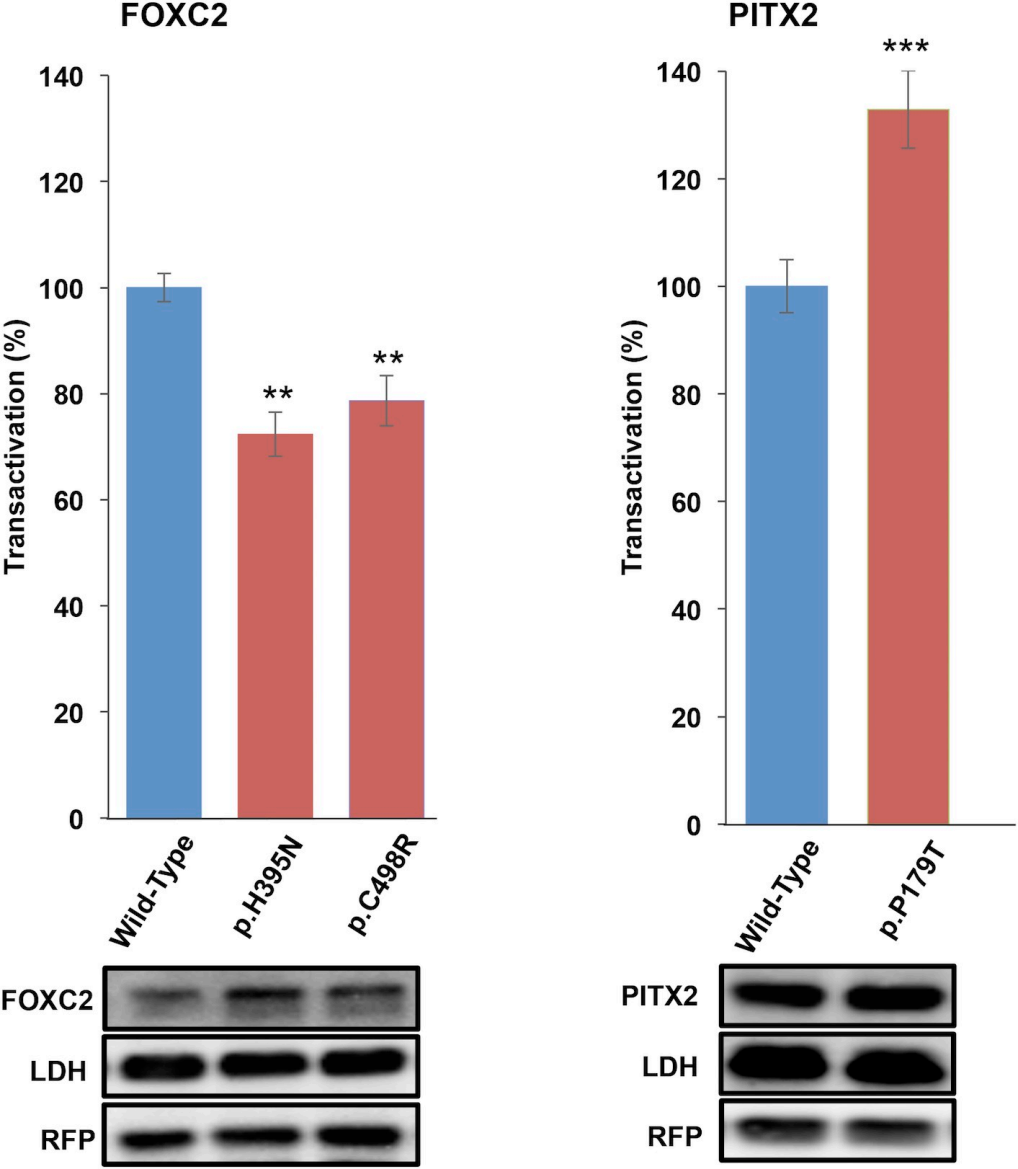
Altered transcriptional activity of FOXC2 and PITX2 variants identified in this study. cDNA constructs encoding the different variants were transiently co-expressed in HEK-293T cells along with cDNAs constructs encoding luciferase coupled to the *CXCR4* promoter (transactivation reporter) and the red fluorescent protein (RFP) as a transfection control. The transcriptional activity was normalized to the amount of the corresponding wild type protein and represented as percentage of the luciferase activity of the wild-type protein. Each lane contained 15 μg of total protein obtained from the cell lysates. The protein levels of the different FOXC2 and PITX2 versions present in HEK-293T cells 24 h after transfection were determined by western immunoblot using a monoclonal anti-myc antibody (1:300) (Santa Cruz). RFP was analyzed via western immunoblot using an anti-RFP antibody (1:500) (Evrogen). The sample loading control, endogenous LDH, was also detected via immunoblot using an anti-LDH antibody (1:1000) (Chemicon). Error bars correspond to the SD of two independent experiments carried out in triplicate. Asterisks indicate statistical significance as compared to the control: P<0,01 (**); P<0,001 (***). Significance was calculated by one-way ANOVA followed by Tukey multiple-comparison test and t-student test.

Transactivation activity of two *FOXC2* and one *PITX2* coding variants was evaluated in HEK-293T cells in culture by transient co-expression of the different variants with luciferase fused to the *CXCR4* proximal promoter, which has been reported to be activated by both FOXC1 and FOXC2 proteins [40]. To the best of our knowledge the CXCR4 promoter has not previously been used to assess PITX2 transactivation. However, we obtained a positive response in the initial assays carried out to set up the functional evaluation of PITX2, and we decided to use this promoter in further experiments. The results showed that the two FOXC2 variants p.(H395N) and p.(C498R) were associated with a 22%-28% reduced transactivation (Fig 5A). Only one of the two identified *PITX2* variants, p.(P179T), could be cloned for functional evaluation. It showed approximately 130% increased transactivation compared with the wild type (Fig 5B). Control Western blot analysis of RFP (transfection control) and LDH (loading control) indicated that these results were not biased by significant loading or transfection differences (Fig 5).

**Fig 6:**
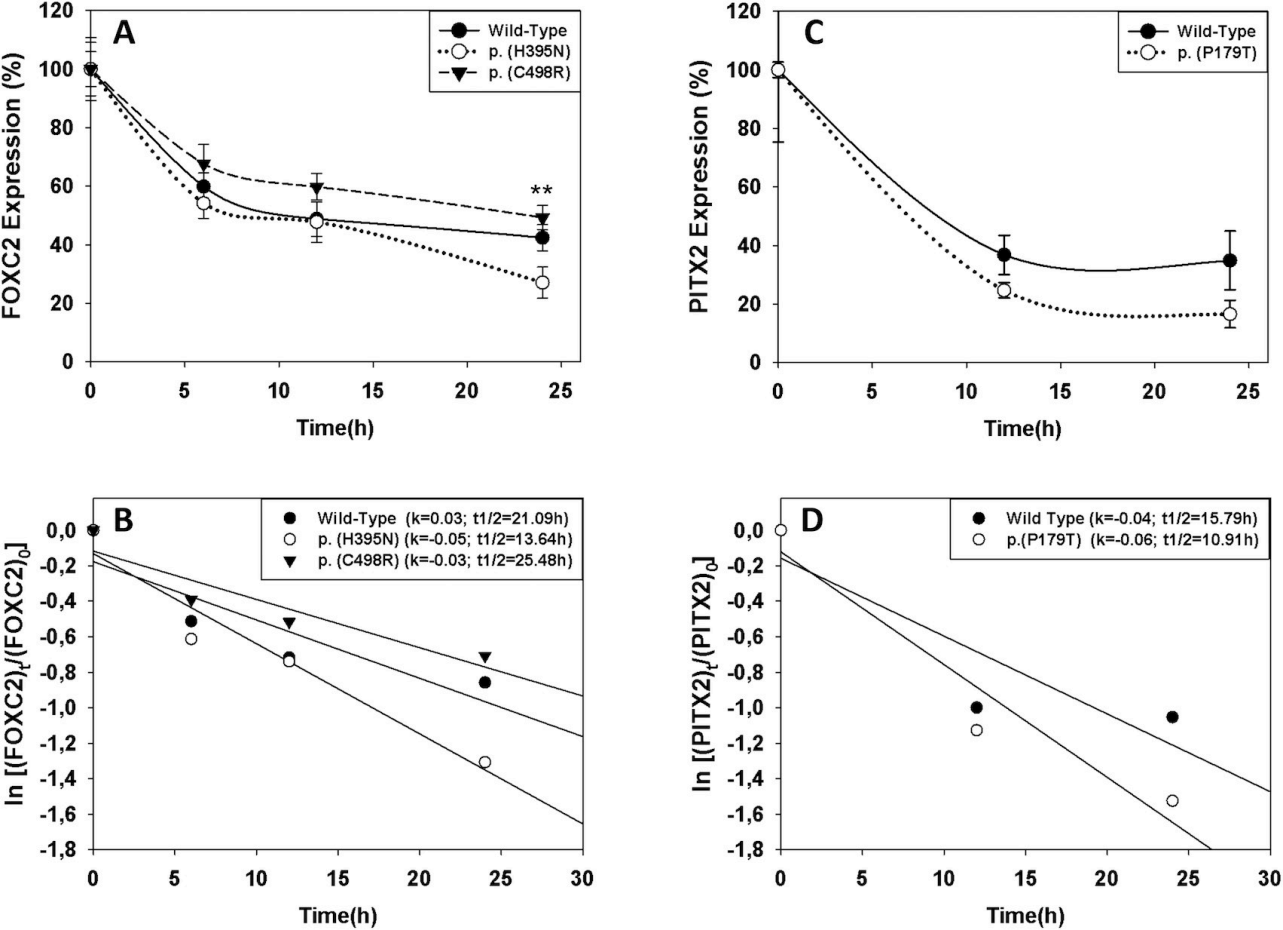
FOXC2 p.H395N variant decreases protein stability. Time course stability analysis of FOXC2 (A) and PITX2 (C) coding variants found in PCG patients was carried out by transient expression in HEK-293T cells. Transfected cells were treated with the protein synthesis inhibitor cycloheximide and the different recombinant proteins were detected by Western immunoblot using an anti-myc monoclonal antibody (Santa Cruz) at the indicated time points, as explained in Materials and Methods. Relative amounts of FOXC2 are expressed as a percentage of levels at time 0 h. The rate of decay and half-lives of the recombinant FOXC2 (B) and PITX2 (D) coding variants at the indicated time-points were determined from linear regression analysis as described in the Materials and Methods. Error bars correspond to the SD of three independent experiments carried out in triplicate. Asterisks indicate statistical significance compared to the control: p<0.01 (**). Two-way ANOVA followed by Tukey multiple-comparison test.

The effect of the *FOXC2* and *PITX2* variants on protein stability was also assessed by transient expression in HEK-293T cells and measurement of recombinant protein levels by Western blot at different times after protein synthesis inhibition with cycloheximide. p.(C498R) stability was not significantly different from that of FOXC2 wild-type protein (Fig 6A), with an estimated half-life of 25.4 h (Fig 6B). These data indicate that p.(C498R) alter transcriptional activity independently of protein stability. However, p.(H395N) showed an approximately 20% reduced stability after 24 h of protein synthesis inhibition (Fig 6A), and a 1.5-fold decreased half-life compared with the wild-type protein (Fig 6B) which may explain the decreased transactivation observed in the previous functional assay. The PITX2 variant p.(P179T) did not show significant protein stability differences with the wild-type protein (Fig 6C and Fig 6D).

**Fig 7:**
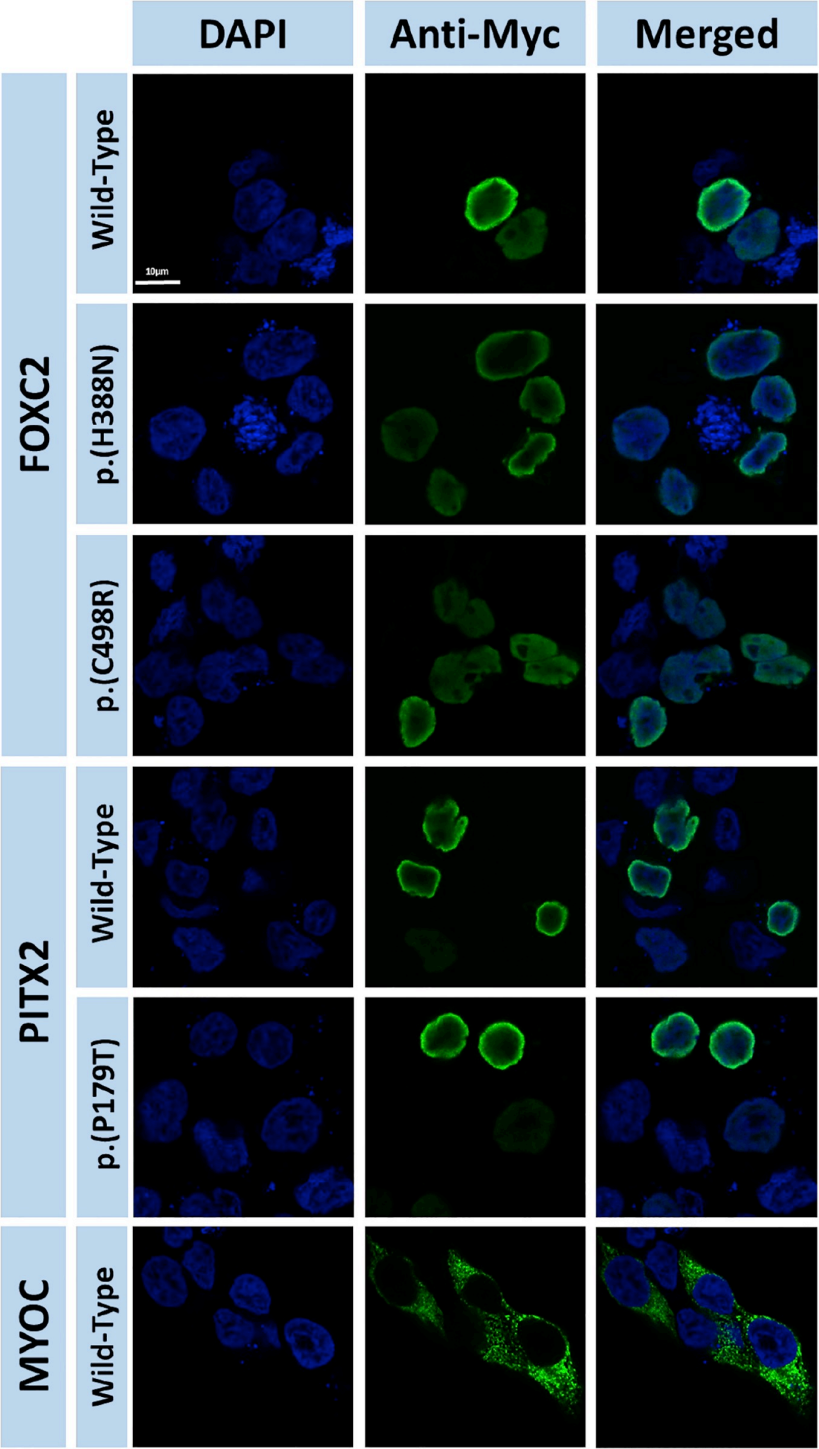
Coding FOXC2 and PITX2C variants identified in this study do not alter subcellular localization in cells in culture. cDNA constructs encoding the indicated recombinant versions of FOXC2 and PITX2C were transiently expressed in HEK-293T cells. The recombinant proteins were detected by fluorescent immunocytochemistry with an anti-myc antibody (Santa Cruz). Nuclei were visualized by fluorescent DAPI staining. Original magnification: X600.

Fluorescence immunocytochemistry of the different recombinant FOXC2 and PITX2 coding variants transiently expressed in HEK-293T cells showed clear nuclear localization. Expression analysis of recombinant myocilin was used as a negative control of nuclear localization, because this protein is known to be absent in the nucleus and present in cytoplasmic secretory vesicles and endoplasmic reticulum and [33]. These analyses demonstrated that the identified coding FOXC2 and PITX2 variants do not affect subcellular localization (Fig 7).

## Discussion

### Elevated frequency of rare *FOXC2 and PITX2* variants in congenital glaucoma patients and evidence of non-Mendelian inheritance

It is accepted that CG is caused by developmental abnormalities of the ocular trabecular meshwork [41]. Herein, we have extended our previous investigations on CG genetics to study the role of *FOXC2* and *PITX2* gene variants in our cohort of patients. As a rationale for these studies we have proposed that different combinations of moderate functional alterations of genes involved in development of the anterior segment of the eye might contribute to development of relatively mild phenotypic alterations, limited to glaucoma, with no other systemic and ocular alterations. This hypothesis implies an oligogenic or polygenic genetic background for this disease, at least in some patients. This and our previous study reveal a higher than expected by chance frequency of rare *FOXC2* and *FOXC1* [19, 20] variants in the analysed cohort of PCG patients (6% and 7.5%, respectively), with either experimentally assessed or inferred moderate functional defects. Segregation analysis of these variants, which were present in the heterozygous state, rule out a monogenic inheritance pattern, indicating according to our earlier reports [19, 20], that these genetic changes might be involved in either oligogenic or complex transmission of the disease. Moreover, the existence of incomplete penetrance, variable expressivity and of a relatively high proportion (close to 20%) of PCG patients with rare heterozygous *CYP1B1* variants [13, 34] also suggest non-Mendelian PCG transmission in some cases. In these patients, disease outcome might depend on modifier factors (genetic, stochastic and/or environmental) [13, 42], as will be discussed later.

### Functional impact of the rare variants

The two missense FOXC2 variants (p.(H395N) and (p.(C498R)) and one of the PITX2 amino acid substitutions (p.(P179T)) were inferred to cause a moderate functional effect at least by one bioinformatic analysis and, experimentally, they were found to be associated with moderately disrupted transactivation. The functional impact of the second PITX2 amino acid substitution, p.(A188T), could not be functionally evaluated due to DNA cloning difficulties. In fact, the two FOXC2 amino acid changes were found to be hypomorphic whereas the PITX2 amino acid substitution (p.(P179T)) behaved experimentally as a hypermorphic variant. Additional structural and functional analysis indicated that p.(H395N) alters polypeptide chain conformation and decreases protein stability, which can explain the associated reduced transactivation. However, our data showed that the transactivation effect of p.(C498R) is not due either to altered protein stability, protein structure or subcellular localization, suggesting that the substitution of a basic (arginine) for a polar (cysteine) amino acid may disrupt protein interactions in the second inhibitory domain of the protein, leading to reduced protein activity. Similarly, our results indicate that increased transactivation associated with the p.P179T PITX2 mutation is not related with altered protein stability, protein conformation or subcellular localization. Proline possesses a hydrophobic side chain, whereas threonine side chain has both hydrophilic and hydrophobic functions. Therefore, this amino acid replacement may affect protein interactions taking place in the transcriptional inhibitory domain where it is located, leading to increased PITX2 activity. In this line, an increased side chain polarity associated with amino acid substitution p.(A188T) could also interfere protein interactions involving the first PITX2 transcriptional inhibitory domain, leading to a functional alteration. Additional studies are required to evaluate these hypotheses. Interestingly, according to Ensembl Regulatory Build, *FOXC2* variants p.S36S (synonymous) and c.*38T>G (non coding 3’ UTR) also mapped at a promoter, which overlapped with *FOXC2* and *FOXC2-AS1* genes. *FOXC2-AS1* encodes a lncRNA transcribed from the negative strand of *FOXC2*. Both molecules have been located in the cytoplasm and are concordantly upregulated in doxorubicin-resistant osteosarcoma cell lines and tissues [43]. It has also been suggested that *FOXC2-AS1* stabilizes *FOXC2* mRNA by forming an RNA-RNA duplex, which subsequently upregulated steady-state FOXC2 expression at both the transcription and post-transcription levels [43]. Therefore, the non-coding nucleotide substitutions and the synonymous variant also have the potential to disrupt gene expression by affecting the promoter. Additionally, p.(S36S) might affect *FOXC2* activity through *FOXC2-AS1* dysregulation. Further studies are required to confirm these ideas. Finally, the high evolutionary conservation of wild type *FOXC2* and *PITX2* nucleotide or amino acid positions involved in the variants identified in this study, further indicate that their substitution by infrequent residues might modify their function.

### Mild functional alterations of *FOXC2* and *PITX2* as modifier factors in PCG

Modifier genes are increasingly recognized as an important source of phenotypic variation that may explain the relationship of phenotype to genotype. Individually, genetic modifiers have small effect size on the phenotypic outcome, leading to reduced penetrance, i.e., not all individuals harbouring a pathogenic variant will exhibit clinical signs of the associated disorder [44]. Normal ocular iridocorneal angle development is the result of highly regulated and intricate gene and protein interactions that can be disrupted by different combinations of subtle functional alteration in multiple genes. In line with this idea, anterior segment abnormalities and CG may manifest in individuals who are heterozygous for functionally altered alleles at multiple genes, resulting in a clinically significant disruption of trabecular meshwork development. This phenomenon is known as synergistic heterozygosity [45]. A representative identified molecular interaction that regulates anterior segment development involves *FOXC1* and *PITX2A*. In fact, it has been reported that PITX2A protein can act as a negative regulator of FOXC1 through formation of PITX2A-FOXC1 heterocomplexes [35]. Therefore, the molecular interaction between these two proteins may affect expression of different target genes. It is also well documented that strict activity levels of these genes are required for normal development of the anterior segment of the eye [29, 46], and that genetic alterations resulting in variable functional disruption (e.g., hypo- and hypermorphic mutations, deletions and duplications of both PITX2 and FOXC1), underlie the phenotypic spectrum of anterior segment associated-diseases [30, 47, 48]. Therefore, activity and/or concentration variations of the individual proteins may influence the phenotypic outcome. The identification of patients in our cohort, as well as in other studies, who combine this type of variants in heterozygosis in two genes (*PITX2*/*CYP1B1*, *PITX2*/*FOXC1* [19], *FOXC1*/*CYP1B1* [49] and *CYP1B1/GJA1* [50]) provide further evidence for this hypothesis. Digenic inheritance involving *CYP1B1* and *MYOC* has been reported in early-onset congenital glaucoma [17] and PCG [16]. Likewise, in this study we have found two glaucoma patients who carried heterozygous rare *PITX2* and *CYP1B1* variants and showed very early glaucoma onset and severe disease, characterised by a large number of surgical operations required to control IOP. These data suggest a modifier role of rare *PITX2* variants on age at onset and severity on *CYP1B1* associated congenital glaucoma. Our data also revealed the presence of one *PITX2 de novo* mutation in one PCG patient, indicating that this type of genetic variants might also be involved in congenital glaucoma, adding additional genetic complexity to the genetic basis of this disease. We should keep in mind that reduced penetrance, low frequency of these variants and limited number of available patients due to low prevalence of the disease, will also complicate studies to determine the role of this type of genetic variation in CG. Combination of genome, transcriptome and even proteome analysis of individual patients, along with functional studies in animal models will be required to gain new insights into the genetic complexity of congenital glaucoma.

In summary, this study further supports the concept that mild functional alterations associated with variants in genes involved in ocular development may act as modifier factors in non-Mendelian CG cases, providing novel insights to understand the genetic complexity of this disease.

## Supporting information

S1 Fig Electropherograms of *FOXC2*, *PITX2* and *CYP1B1* variants identified in this study. Arrows over the peaks indicate the location of mutations. The mutant nucleotides are indicated in red and those in heterozygosis are shown below the corresponding wild type position. The sequences of the different variants were obtained from the corresponding probands in each family and the wild type sequences from control subjects.
(TIF)

S1 Table The primer sequences and PCR conditions used for *FOXC2* gene sequencing. (DOCX)

## References

[1] Chan TCP, Brookes J, Cavuoto K, Bitrian E, Grajewski AL. Primary congenital glaucoma and juvenile open-angle glaucoma In: Weinreb RN, Grajewski AL, Papadopoulos M, Grigg J, Freedman S, editors. Childhood Glaucoma. Ámsterdam, The Netherlands: Kluger Publications; 2013 p. 137–53.

[2] RN S, DI W. Infa ntile glaucoma: diagnosis and differential diagnosis Congenital and Pediatric Glaucomas. St. Louis: CV Mosby; 1970 p. 37–59.

[3] Francois J. Congenital glaucoma and its inheritance. Ophthalmologica. 1980;181(2):61–73. 10.1159/000309028 7219964

[4] Gencik A. Epidemiology and genetics of primary congenital glaucoma in Slovakia. Description of a form of primary congenital glaucoma in gypsies with autosomal-recessive inheritance and complete penetrance. Dev Ophthalmol. 1989;16:76–115. 2676634

[5] Elder MJ. Congenital glaucoma in the West Bank and Gaza Strip. Br J Ophthalmol. 1993;77(7):413–6. 8343468 10.1136/bjo.77.7.413 PMC504551

[6] Sarfarazi M, Stoilov I. Molecular genetics of primary congenital glaucoma. Eye. 2000;14 (Pt 3B):422–8. 11026969 10.1038/eye.2000.126

[7] Stoilov I, Akarsu AN, Sarfarazi M. Identification of three different truncating mutations in cytochrome P4501B1 (CYP1B1) as the principal cause of primary congenital glaucoma (Buphthalmos) in families linked to the GLC3A locus on chromosome 2p21. Hum Mol Genet. 1997;6(4):641–7. 9097971 10.1093/hmg/6.4.641

[8] Ali M, McKibbin M, Booth A, Parry DA, Jain P, Riazuddin SA, et al Null mutations in LTBP2 cause primary congenital glaucoma. AmJ Hum Genet. 2009;84(5):664–71. 19361779 10.1016/j.ajhg.2009.03.017 PMC2680998

[9] Souma T, Tompson SW, Thomson BR, Siggs OM, Kizhatil K, Yamaguchi S, et al Angiopoietin receptor TEK mutations underlie primary congenital glaucoma with variable expressivity. J Clin Invest. 2016;126(7):2575–87. 10.1172/JCI85830 27270174 PMC4922711

[10] Ferre-Fernández JJ, Aroca-Aguilar JD, Medina-Trillo C, Bonet-Fernández JM, Méndez-Hernández CD, Morales-Fernández L, et al Whole-Exome Sequencing of Congenital Glaucoma Patients Reveals Hypermorphic Variants in GPATCH3, a New Gene Involved in Ocular and Craniofacial Development. Sci Rep. 2017;7:46175 Epub 2017/04/11. 10.1038/srep46175 28397860 PMC5387416

[11] Mashima Y, Suzuki Y, Sergeev Y, Ohtake Y, Tanino T, Kimura I, et al Novel cytochrome P4501B1 (CYP1B1) gene mutations in Japanese patients with primary congenital glaucoma. Invest Ophthalmol Vis Sci. 2001;42(10):2211–6. 11527932

[12] Bagiyeva S, Marfany G, Gonzalez-Angulo O, Gonzalez-Duarte R. Mutational screening of CYP1B1 in Turkish PCG families and functional analyses of newly detected mutations. Mol Vis. 2007;13:1458–68. 17893647

[13] López-Garrido M-P, Medina-Trillo C, Morales-Fernandez L, Garcia-Feijoo J, Martínez-De-La-Casa J-M, García-Antón M, et al Null CYP1B1 genotypes in primary congenital and nondominant juvenile glaucoma. Ophthalmology. 2013;120(4):716–23. 10.1016/j.ophtha.2012.09.016 23218183

[14] Narooie-Nejad M, Paylakhi SH, Shojaee S, Fazlali Z, Rezaei KM, Nilforushan N, et al Loss of function mutations in the gene encoding latent transforming growth factor beta binding protein 2, LTBP2, cause primary congenital glaucoma. Hum Mol Genet. 2009;18(20):3969–77. 10.1093/hmg/ddp338 19656777

[15] Azmanov DN, Dimitrova S, Florez L, Cherninkova S, Draganov D, Morar B, et al LTBP2 and CYP1B1 mutations and associated ocular phenotypes in the Roma/Gypsy founder population. Eur J Hum Genet. 2011;19(3):326–33. 10.1038/ejhg.2010.181 21081970 PMC3062003

[16] Kaur K, Reddy AB, Mukhopadhyay A, Mandal AK, Hasnain SE, Ray K, et al Myocilin gene implicated in primary congenital glaucoma. Clin Genet. 2005;67(4):335–40. 10.1111/j.1399-0004.2005.00411.x 15733270

[17] Vincent AL, Billingsley G, Buys Y, Levin AV, Priston M, Trope G, et al Digenic inheritance of early-onset glaucoma: CYP1B1, a potential modifier gene. Am J Hum Genet. 2002;70(2):448–60. 10.1086/338709 11774072 PMC384919

[18] Medina-Trillo C, Sánchez-Sánchez F, Aroca-Aguilar JD, Ferre-Fernández JJ, Morales L, Méndez-Hernández CD, et al Hypo- and hypermorphic FOXC1 mutations in dominant glaucoma: transactivation and phenotypic variability. PLoS One. 2015;10(3):e0119272 10.1371/journal.pone.0119272 25786029 PMC4364892

[19] Medina-Trillo C, Aroca-Aguilar J-D, Mendez-Hernandez C-D, Morales L, Garcia-Anton M, Garcia-Feijoo J, et al Rare FOXC1 variants in congenital glaucoma: identification of translation regulatory sequences. Eur J Hum Genet. 2016;24(5):672–80. 10.1038/ejhg.2015.169 26220699 PMC4930079

[20] Medina-Trillo C, Aroca-Aguilar JD, Ferre-Fernández JJ, Méndez-Hernández CD, Morales L, García-Feijoo J, et al The Role of hsa-miR-548l Dysregulation as a Putative Modifier Factor for Glaucoma-Associated FOXC1 Mutations. Microrna. 2015;4(1):50–6. 25809640 10.2174/2211536604666150320234654

[21] Acharya M, Huang L, Fleisch VC, Allison WT, Walter MA. A complex regulatory network of transcription factors critical for ocular development and disease. Hum Mol Genet. 2011;20(8):1610–24. 10.1093/hmg/ddr038 21282189

[22] Lehmann OJ, Sowden JC, Carlsson P, Jordan T, Bhattacharya SS. Fox's in development and disease. Trends Genet. 2003;19(6):339–44. 10.1016/S0168-9525(03)00111-2 12801727

[23] Carlsson P, Mahlapuu M. Forkhead transcription factors: key players in development and metabolism. Dev Biol. 2002;250(1):1–23. 12297093 10.1006/dbio.2002.0780

[24] Berry FB, Tamimi Y, Carle MV, Lehmann OJ, Walter MA. The establishment of a predictive mutational model of the forkhead domain through the analyses of FOXC2 missense mutations identified in patients with hereditary lymphedema with distichiasis. Hum Mol Genet. 2005;14(18):2619–27. Epub 2005/08/04. 10.1093/hmg/ddi295 16081467

[25] Winnier GE, Hargett L, Hogan BL. The winged helix transcription factor MFH1 is required for proliferation and patterning of paraxial mesoderm in the mouse embryo. Genes Dev. 1997;11(7):926–40. 9106663 10.1101/gad.11.7.926

[26] Hiemisch H, Monaghan AP, Schütz G, Kaestner KH. Expression of the mouse Fkh1/Mf1 and Mfh1 genes in late gestation embryos is restricted to mesoderm derivatives. Mech Dev. 1998;73(1):129–32. 9545561 10.1016/s0925-4773(98)00039-2

[27] Kume T, Jiang H, Topczewska JM, Hogan BL. The murine winged helix transcription factors, Foxc1 and Foxc2, are both required for cardiovascular development and somitogenesis. Genes Dev. 2001;15(18):2470–82. 10.1101/gad.907301 11562355 PMC312788

[28] Fang J, Dagenais SL, Erickson RP, Arlt MF, Glynn MW, Gorski JL, et al Mutations in FOXC2 (MFH-1), a forkhead family transcription factor, are responsible for the hereditary lymphedema-distichiasis syndrome. Am J Hum Genet. 2000;67(6):1382–8. 10.1086/316915 11078474 PMC1287915

[29] Semina EV, Reiter R, Leysens NJ, Alward WL, Small KW, Datson NA, et al Cloning and characterization of a novel bicoid-related homeobox transcription factor gene, RIEG, involved in Rieger syndrome. Nat Genet. 1996;14(4):392–9. 10.1038/ng1296-392 8944018

[30] Seifi M, Walter MA. Axenfeld-Rieger syndrome. Clin Genet. 2017 Epub 2017/10/03. 10.1111/cge.13148 28972279

[31] Cox CJ, Espinoza HM, McWilliams B, Chappell K, Morton L, Hjalt TA, et al Differential regulation of gene expression by PITX2 isoforms. J Biol Chem. 2002;277(28):25001–10. Epub 2002/04/10. 10.1074/jbc.M201737200 11948188

[32] Ceroni F, Aguilera-Garcia D, Chassaing N, Bax DA, Blanco-Kelly F, Ramos P, et al New GJA8 variants and phenotypes highlight its critical role in a broad spectrum of eye anomalies. Hum Genet. 2018 Epub 2018/02/20. 10.1007/s00439-018-1875-2 29464339

[33] Sanchez-Sanchez F, Martinez-Redondo F, Aroca-Aguilar JD, Coca-Prados M, Escribano J. Characterization of the intracellular proteolytic cleavage of myocilin and identification of calpain II as a myocilin-processing protease. J Biol Chem. 2007;282(38):27810–24. 10.1074/jbc.M609608200 17650508

[34] Campos-Mollo E, Lopez-Garrido MP, Blanco-Marchite C, Garcia-Feijoo J, Peralta J, Belmonte-Mart¡nez J, et al CYP1B1 gene mutations in Spanish patients with primary congenital glaucoma: phenotypic and functional variability. Mol Vis. 2009;15:417–31. 19234632 PMC2645906

[35] Berry FB, Lines MA, Oas JM, Footz T, Underhill DA, Gage PJ, et al Functional interactions between FOXC1 and PITX2 underlie the sensitivity to FOXC1 gene dose in Axenfeld-Rieger syndrome and anterior segment dysgenesis. Hum Mol Genet. 2006;15(6):905–19. 10.1093/hmg/ddl008 16449236

[36] Aroca-Aguilar J-D, Martinez-Redondo F, Martin-Gil A, Pintor J, Coca-Prados M, Escribano J. Bicarbonate-Dependent Secretion and Proteolytic Processing of Recombinant Myocilin. Plos One. 2013;8(1). 10.1371/journal.pone.0054385 23342144 PMC3547000

[37] Xu D, Zhang Y. Toward optimal fragment generations for ab initio protein structure assembly. Proteins. 2013;81(2):229–39. Epub 2012/10/16. 10.1002/prot.24179 ; 22972754 PMC3551984

[38] McLaren W, Gil L, Hunt SE, Riat HS, Ritchie GR, Thormann A, et al The Ensembl Variant Effect Predictor. Genome Biol. 2016;17(1):122 Epub 2016/06/06. 10.1186/s13059-016-0974-4 27268795 PMC4893825

[39] Furukawa T, Kozak CA, Cepko CL. rax, a novel paired-type homeobox gene, shows expression in the anterior neural fold and developing retina. Proc Natl Acad Sci U S A. 1997;94(7):3088–93. 9096350 10.1073/pnas.94.7.3088 PMC20326

[40] Hayashi H, Kume T. Forkhead transcription factors regulate expression of the chemokine receptor CXCR4 in endothelial cells and CXCL12-induced cell migration. Biochem Biophys Res Commun. 2008;367(3):584–9. 10.1016/j.bbrc.2007.12.183 18187037 PMC2265419

[41] Sampaolesi R., Zarate J., Sampaolesi J.R. Primary Congenital Glaucoma The Glaucomas. 1 Pediatric Glaucomas. Heidelberg: Springer-Verlag; 2009 p. 1–7.

[42] Campos-Mollo E, Lopez-Garrido M-P, Blanco-Marchite C, Garcia-Feijoo J, Peralta J, Belmonte-Martinez J, et al CYP1B1 mutations in Spanish patients with primary congenital glaucoma: phenotypic and functional variability. Mol Vis. 2009;15(42):417–31. 19234632 PMC2645906

[43] Zhang CL, Zhu KP, Ma XL. Antisense lncRNA FOXC2-AS1 promotes doxorubicin resistance in osteosarcoma by increasing the expression of FOXC2. Cancer Lett. 2017;396:66–75. Epub 2017/03/16. 10.1016/j.canlet.2017.03.018 28323030

[44] Cooper DN, Krawczak M, Polychronakos C, Tyler-Smith C, Kehrer-Sawatzki H. Where genotype is not predictive of phenotype: towards an understanding of the molecular basis of reduced penetrance in human inherited disease. Hum Genet. 2013;132(10):1077–130. 10.1007/s00439-013-1331-2 ; 23820649 PMC3778950

[45] Dipple KM, Phelan JK, McCabe ER. Consequences of complexity within biological networks: robustness and health, or vulnerability and disease. Mol Genet Metab. 2001;74(1–2):45–50. 10.1006/mgme.2001.3227 11592802

[46] Mears AJ, Jordan T, Mirzayans F, Dubois S, Kume T, Parlee M, et al Mutations of the forkhead/winged-helix gene, FKHL7, in patients with Axenfeld-Rieger anomaly. Am J Hum Genet. 1998;63(5):1316–28. 10.1086/302109 9792859 PMC1377542

[47] Kozlowski K, Walter MA. Variation in residual PITX2 activity underlies the phenotypic spectrum of anterior segment developmental disorders. HumMolGenet. 2000;9(14):2131–9. 10.1093/hmg/9.14.2131 10958652

[48] D'Haene B, Meire F, Claerhout I, Kroes HY, Plomp A, Arens YH, et al Expanding the spectrum of FOXC1 and PITX2 mutations and copy number changes in patients with anterior segment malformations. Invest OphthalmolVisSci. 2011;52(1):324–33. 10.1167/iovs.10-5309 20881294

[49] Chakrabarti S, Kaur K, Rao KN, Mandal AK, Kaur I, Parikh RS, et al The transcription factor gene FOXC1 exhibits a limited role in primary congenital glaucoma. Invest OphthalmolVisSci. 2009;50(1):75–83. 10.1167/iovs.08-2253 18708620

[50] Cella W, de Vasconcellos JP, de Melo MB, Kneipp B, Costa FF, Longui CA, et al Structural assessment of PITX2, FOXC1, CYP1B1, and GJA1 genes in patients with Axenfeld-Rieger syndrome with developmental glaucoma. Invest OphthalmolVisSci. 2006;47(5):1803–9. 10.1167/iovs.05-0979 16638984

