# Supplementary figures and images for: Role of FOXC2 and PITX2 rare variants associated with mild functional alterations as modifier factors in congenital glaucoma
Source: PLoS One. 2019 Jan 18;14(1):e0211029. doi: 10.1371/journal.pone.0211029 (PMC6338360; doi:10.1371/journal.pone.0211029)

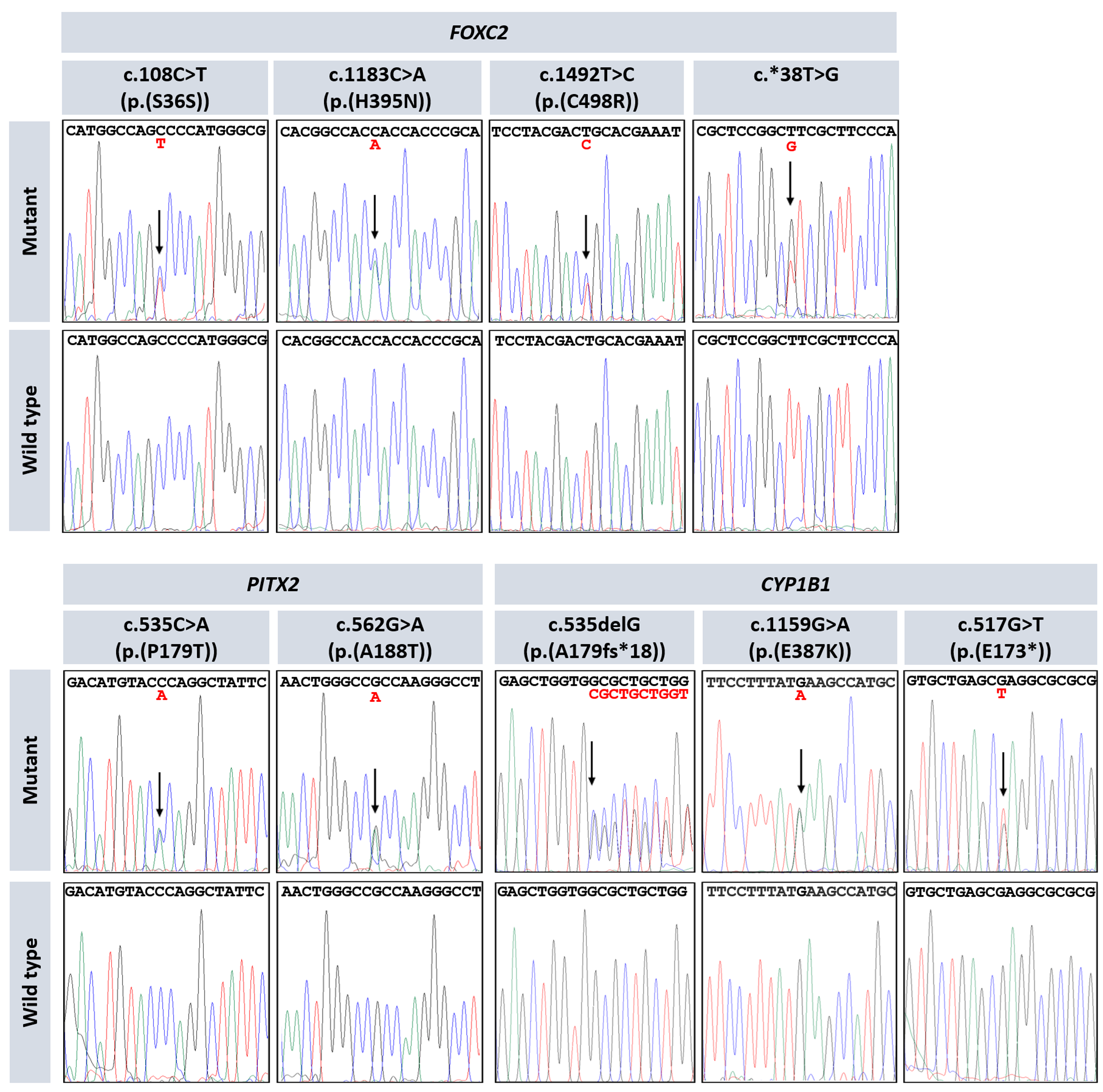

Supplement: S1 Fig — Arrows over the peaks indicate the location of mutations. The mutant nucleotides are indicated in red and those in heterozygosis are shown below the corresponding wild type position. The sequences of the different variants were obtained from the corresponding probands in each family and the wild type sequences from control subjects. (TIF) [file pone.0211029.s001.tif]
