# Supplementary material for: Role of FOXC2 and PITX2 rare variants associated with mild functional alterations as modifier factors in congenital glaucoma
Source: PLoS One. 2019 Jan 18;14(1):e0211029. doi: 10.1371/journal.pone.0211029 (PMC6338360; doi:10.1371/journal.pone.0211029)
Supplement: S1 Table — (DOCX) [file pone.0211029.s002.docx]

**S1 Table. The primer sequences and PCR conditions used for *FOXC2* gene sequencing**.

| **Primer set** | **Sequence (5’→3’)** | **Annealing temperature (ºC)/time (s)** | **PCR cycles** | **DMSO (%)** | **Gene región/amplicon size (bp)** |
| --- | --- | --- | --- | --- | --- |
| A | F: GCCCTAGGGTCGGTGTTAGC | 61/30 | 40 | 0 | PR/660 |
|  | R: TCCTAATTATGGGGGCCCAG |  |  |  |  |
| B | F: CCCCGAGCCTGGAAACTC | 64/30 | 40 | 0 | PR/728 |
|  | R: GCGGACCCAGCGGAG |  |  |  |  |
| C | F: GGGCCGAGGGTCCACC | 65/30 | 40 | 10 | PR-5’UTR-CR/573 |
|  | R: GGCTTCACCAGGTCCTTAGGC |  |  |  |  |
| D | F: TCCTACGCGCCCTACCAC | 62/30 | 40 | 10 | CR/663 |
|  | R: TCGCAGGGTCATGATGTTCTC |  |  |  |  |
| E | F: CCGACGGCTCGCTGC | 65/30 | 40 | 10 | CR/546 |
|  | R: CGGGGAGGTGGTTCAGG |  |  |  |  |
| F | F: CACGTCGCCCCTGAGC | 60/30 | 40 | 10 | CR-3’UTR/635 |
|  | R: TTGGAGGGGTTTCCTGGG |  |  |  |  |

CR: coding region; F: forward; R: reverse; PR: promoter.
